# Impact of Normalization on Entropy-Based Weights in Hellwig’s Method: A Case Study on Evaluating Sustainable Development in the Education Area

**DOI:** 10.3390/e26050365

**Published:** 2024-04-26

**Authors:** Ewa Roszkowska, Tomasz Wachowicz

**Affiliations:** 1 Faculty of Computer Science, Bialystok University of Technology, Wiejska 45A, 15-351 Bialystok, Poland; 2 Department of Operations Research, University of Economics in Katowice, 1 Maja 50, 40-287 Katowice, Poland; tomasz.wachowicz@uekat.pl

**Keywords:** MCDM, entropy-based weights method, normalization, Hellwig’s method, sustainable development, education

## Abstract

Determining criteria weights plays a crucial role in multi-criteria decision analyses. Entropy is a significant measure in information science, and several multi-criteria decision-making methods utilize the entropy weight method (EWM). In the literature, two approaches for determining the entropy weight method can be found. One involves normalization before calculating the entropy values, while the second does not. This paper investigates the normalization effect for entropy-based weights and Hellwig’s method. To compare the influence of various normalization methods in both the EWM and Hellwig’s method, a study evaluating the sustainable development of EU countries in the education area in the year 2021 was analyzed. The study used data from Eurostat related to European countries’ realization of the SDG 4 goal. It is observed that vector normalization and sum normalization did not change the entropy-based weights. In the case study, the max–min normalization influenced EWM weights. At the same time, these weights had only a very weak impact on the final rankings of countries with respect to achieving the SDG 4 goal, as determined by Hellwig’s method. The results are compared with the outcome obtained by Hellwig’s method with equal weights. The simulation study was conducted by modifying Eurostat data to investigate how the different normalization relationships discovered among the criteria affect entropy-based weights and Hellwig’s method results.

## 1. Introduction

Multiple criteria decision-making (MCDM) has evolved as a crucial component of operations research, focusing on developing mathematical tools to facilitate the subjective evaluation of performance criteria by decision-makers [1]. MCDM techniques address situations where decisions involve multiple, often conflicting, criteria or objectives. These methods help decision-makers assess and prioritize various alternatives based on different criteria, taking into account the inherent complexity and subjectivity of decision-making processes. Several approaches within MCDM have been investigated, each customized to suit the specific decision contexts and preferences of decision-makers [1,2,3].

The weights assigned by decision makers (DMs) to differentiate the importance of criteria play a pivotal role in the multi-criteria decision-making process. Numerous methods exist for determining these weights for DMs [4,5,6] that can be classified into two categories of approaches [7,8]: subjective and objective. The subjective approach relies on evaluations provided by DMs, whereas the objective approach relies on intrinsic information contained in the dataset describing the criteria performances. Various subjective methods are available, including the analytic hierarchy process (AHP) [9,10], rank-based [11,12], direct rating [13,14], Delphi method [15], and point allocation methods [13,14]. On the other hand, examples of objective approaches include CRiteria Importance Through Inter-criteria Correlation (CRITIC) [16], standard deviation (SD), and the entropy weight method (EWM) [15]. Among methods for determining objective weights, the EWM is widely adopted and particularly valuable in decision-making processes, especially when there is a lack of prior knowledge about the relative importance of criteria. In the literature, there are two approaches to determining the EWM: one involves normalizing the decision matrix before calculating the entropy value for each criterion, and the other calculates the entropy value directly from the decision matrix [17]. The first approach commonly employs the max–min normalization method.

Let us note that normalization stands as a crucial stage in most MCDM methods, aligning all criteria onto a uniform scale and enabling a comparison among alternatives. Several papers argue the importance of the choice of normalization techniques and their impact on different rankings of alternatives [18,19,20]. Although two variants of entropy-based weights are frequently used in the research (see Section 2.2), only studies [17,21] analyzed the effects of normalization in the EWM on TOPSIS. Therefore, it is vital to analyze the effects of normalization in the EWM using other multi-criteria techniques.

This paper addresses the impact of normalization on entropy weights and the resulting rank ordering in Hellwig’s method. Hellwig’s method is a multi-criteria decision-making technique that facilitates ranking alternatives based on their proximity to the ideal solution [22,23]. In the classical Hellwig’s approach, standardization (S) is used based on mean and standard deviations from the set of observations to handle performances measured by different scales before determining the distances. However, in some studies, S was replaced by max–min normalization or vector normalization. Therefore, it would also be vital to analyze the effects of different normalization methods on Hellwig’s approach to rank-ordering alternatives.

We use the problem of evaluating sustainable development in the education of EU countries based on real-world Eurostat data to show the influence of various normalization methods on entropy-based weights and Hellwig-based rankings. Surprisingly, the results in our specific decision-making context show that despite having a potential impact on significant differences in the determination of weights, they may only marginally influence the final rankings. Therefore, the simulation study was conducted to verify if these results may have a more general interpretation when the decision-making context changes. In a series of replications, we modified Eurostat data to investigate and discuss the compatibility between entropy-based weights and Hellwig’s method.

The objectives and contributions of this study are as follows:

- Compare the performance of two variants of entropy-based weight methods in assessing sustainable development in education.
- Evaluate the effectiveness of three normalization formulas in Hellwig’s method for assessing sustainable development in education.
- Investigate and compare the combined performance of entropy-based weight methods and normalization within Hellwig’s method for assessing sustainable development in education.
- Conduct the simulation study by modifying Eurostat data to discuss and investigate the sensitivity of the obtained results and provide more general conclusions regarding the influence of normalization on entropy-based weights in Hellwig’s approach.

The rest of the paper is structured as follows: Section 2 introduces the concept of the EWM and a short literature review concerning the application of entropy weights in decision-making. Section 3 introduces Hellwig’s method. Section 4 presents the results, and Section 5 discusses the findings from the simulation study. Finally, Section 6 presents the conclusions.

## 2. The Preliminaries and Literature Review

This section introduces the concept of the entropy-based weight method and presents related work that encompasses the EWM in decision-making problems.

### 2.1. Entropy-Based Weight Method

The concept of entropy, originally developed by Claude Shannon [24] in his seminal paper titled ‘A Mathematical Theory of Communication’ published in 1948, has become a significant measure widely used in information theory. In information theory, entropy measures the uncertainty or randomness associated with a random variable. It quantifies the average amount of information required to describe the outcomes of a random process. The higher the entropy, the greater the uncertainty.

In decision theory, entropy is often used to assess the uncertainty or information content of different alternatives, particularly in determining the weight of criteria in the MCDM process. The decision matrix contains a certain amount of information. Since each column of this matrix describes a single-criterion performance of alternatives, the EWM may allow for the objective calculation of weights based on differences in amounts of information ensured by each criterion. Thereby, the impact of subjective judgments is minimized [25,26,27,28]. For instance, a criterion has less influence when all alternatives share similar values for that specific criterion. Additionally, if all values are the same, it becomes possible to eliminate that attribute from consideration [15]. In the literature, two variants of the entropy-based weight method are presented. The first variant of the EWM involves no normalization, while the second one includes normalization, usually max–min, before calculating the entropy value for each criterion [17].

Let us assume that we have *m* alternatives $A_{1},A_{2},\ldots ,A_{m}$ and *n* decision criteria $C_{1},C_{2},\ldots ,C_{n}$. The general framework for calculating the EWM in multiple-criteria decision-making is outlined as follows:

Step 1. Determination of decision matrix.

The decision matrix *D* has the form:

(1) $$D=\left[x_{ij}\right]\;$$

where $x_{ij}$ is the value of the j-th criterion for the i-th alternative i=1, 2,…, m, j=1, 2,…, n.

Step 2 (optional). Normalization of decision matrix.

A normalized decision matrix has the form:

(2) $$Z=\left[z_{ij}\right]\;$$

where $z_{ij}$ is the normalized value $x_{ij}$ of the j-th criterion for the i-th alternative i=1, 2,…, m, j=1, 2,…, n.

Step 3. Calculation of the information entropy of each criterion.

The information entropy $E_{j}$ for the *j*-th criterion is calculated by the following equation:

(3) $$E_{j}=-\frac{1}{\ln m}\sum_{i=1}^{m}p_{ij}\ln p_{ij},\;j=1,2,\ldots ,n.$$

where

(4) $$p_{ij}=\frac{x_{ij}}{\sum_{i}^{m}x_{ij}}\mathrm{for}\mathrm{the}\mathrm{EWM}\mathrm{without}\mathrm{normalization},i.e.,\mathrm{Step}2\mathrm{is}\mathrm{omitted},$$

or

(5) $$p_{ij}=\frac{z_{ij}}{\sum_{i}^{m}z_{ij}}\mathrm{for}\mathrm{the}\mathrm{EWM}\mathrm{with}\mathrm{normalization}\mathrm{in}\mathrm{Step}2.$$

In particular, when $x_{ij}=0$ (or $z_{ij}=0$), then it is assumed that $p_{ij}\ln p_{ij}=0$ for convenience in calculations. To avoid $x_{ij}=0$ or $z_{ij}=0$, Zhu et al. [29] proposed the following modified formula:

(6) $$p_{ij}=\frac{x_{ij}+C}{\sum_{i}^{m}(x_{ij\;}+C)}\;(\mathrm{or}\;p_{ij}=\frac{z_{ij}+C}{\sum_{i}^{m}(z_{ij\;}+C)})$$

where C is a constant that should at least satisfy $x_{ij}+C>0$ ($z_{ij}+C>0).$

Step 4. Calculation of weights.

The weight of the j-th criterion is calculated by the following equation:

(7) $$w_{j}=\frac{1-E_{j}}{\sum_{j=1}^{n}\left(1-E_{j}\right)}=\frac{1-E_{j}}{n-\sum_{j=1}^{n}E_{j}},\;j=1,2,\ldots ,n,$$

where $E_{j}$ is an extended and normalized information entropy calculated using Formula (3).

It is easy to check that $0\;\leq w_{j}\;\leq 1\;\left(j=1,\ldots ,\;n\right)$ and $\sum_{j=1}^{n}w_{j}=1$, according to the properties of entropy.

The lower the information entropy $E_{j}$, the higher the weight j. In other words, the higher the entropy value of $1-E_{j}$, the greater the weight assigned to the j-th criterion. Increased entropy values $1-E_{j}$ signify heightened uncertainty, resulting in a greater weight assigned to the criterion as it holds more decision-relevant information. Conversely, decreased entropy indicates a more predictable criterion, leading to a lower weight. Hence, entropy offers an objective approach to establishing criterion weights. Tackling uncertainty through entropy enhances the robustness of decision-making, especially in scenarios with incomplete or ambiguous information.

The sum method (SM) and vector normalization (VN) are two frequently used normalization formulas in decision-making methods. The calculation equations of the sum method (SM) and vector normalization (VN) are as follows [15]:

(8) $$z_{ij}=\frac{x_{ij}}{\sum_{i=1}^{m}x_{ij}}(\mathrm{sum}\mathrm{method})$$

(9) $$z_{ij}=\frac{x_{ij}}{\sqrt{\sum_{i=1}^{m}{x_{ij}}^{2}}}(\mathrm{vector}\mathrm{normalization}).$$

We can verify that the SM and VN will not alter the entropy-based weights [17]; therefore, there is no point in using them when calculating the EWM in Step 2. It is easy to verify with:

(10) pij=zij∑imzij=xij∑imxij ∑imxij∑imxij=xij∑imxijfortheSMnormalization

and

(11) pij=zij∑imzij=xij∑i=1mxij2 ∑imxij∑i=1mxij2=xij∑imxij forthevectornormalization.

According to the literature gathered in Section 2.2, the max–min (MM) normalization formula is the most commonly employed in the EWM in Step 2. The calculation equation for the MM method is as follows:

(12) $$z_{ij}=\left\{\begin{array}{c} \frac{x_{ij}-\underset{i}{\min}x_{ij}}{\underset{i}{\max}x_{ij}-\underset{i}{\min}x_{ij}}\;\mathrm{for}\mathrm{benefit}\mathrm{criterion}\\ \frac{\underset{i}{\max}x_{ij}-x_{ij}}{\underset{i}{\max}x_{ij}-\underset{i}{\min}x_{ij}}\;\mathrm{for}\mathrm{cost}\mathrm{criterion} \end{array}\right.\;(\max–\min)$$

Therefore, in further analyses, we will concentrate on two variants of the EWM: one without normalization (EWMn) and the other with MM normalization (EWMM) before calculating the entropy value of each criterion.

### 2.2. Literature Review

The entropy-based weight method is a technique widely utilized in MCDM for assessing the relative importance of criteria [15,21,29,30,31]. The effect of normalization on the entropy-based TOPSIS method has been analyzed by Chen [17]. Numerous studies have explored and applied the EWM in various fields, such as management [32], finance [33], environmental quality [34], sustainable energy [8,27], water resources management [6], location selection [35], urban air quality [36], and tourism [8,37].

Dong et al. [32] investigated the risk assessment of water security during drought periods using entropy-weighted methods. Zhang et al. [28] applied TOPSIS and entropy-based weights to evaluate the competitiveness of tourism destinations. Zhang and Wang [38] employed an entropy-weight approach to assess Chongqing’s water resource security between 2000 and 2011. This evaluation aimed to identify the origins of pressure on the water resources system and gauge the effectiveness of current response measures. Wu et al. [39] investigated the sensitivity of entropy-based weights for assessing water quality, employing large stochastic samples in their study. Ding et al. [40] presented a comprehensive evaluation of urban sustainable development in China based on the TOPSIS method with entropy-based weights. Zeng and Huang [41] proposed a synthetic assessment and analysis method incorporating nine risk indices guided by natural disaster risk assessment principles. The AHP method was combined with entropy theory to calculate the weights of indicators that integrated subjective and objective weights. Xu et al. [42] proposed an integrated methodology by incorporating an urban flood inundation model, an improved entropy weight method, and a k-means cluster algorithm to evaluate urban flood risk. The weights were calculated by integrating the entropy weight method and the analytic hierarchy process (AHP) method. Shen and Liao [43] utilized the AHP and the entropy method to develop a risk evaluation model for the food cold chain. Mukhametzyanov [26] conducted a comparative analysis of three objective methods for determining criteria weights in multi-criteria decision-making. The methods examined were entropy, CRITIC, and standard deviation, and various propositions for the aggregation of weights were presented. The common feature of the studies mentioned above is applying the max–min method to the EWM determination in Step 2.

At the same time, in a series of papers, Step 2 has been omitted from the EWM calculation [33,34,44,45]. Aras et al. [33] assessed Garanti Bank’s corporate sustainability performance by examining economic, social, and environmental factors using TOPSIS with an entropy-based weighting method. Dang and Dang [34] assessed the environmental quality of the Organization for Economic Co-operation and Development (OECD) countries using the VIKOR method. The weights of the criteria were determined through the entropy weight method. Tian [45] incorporated the EWM into TOPSIS to evaluate corporate internal control. Hafezalkotob and Hafezalkotob [44] proposed the MULTIMOORA technique, a form of the comprehensive multi-objective optimization based on the ratio analysis (MORRA) technique, with incorporated entropy-based weights for the analysis of the material selection process.

He et al. [25] introduced a method for determining weights and aggregating models in multi-group decision-making. They utilized the entropy weighting technique and the principle of minimum cross-entropy in their approach. Yue [31] applied entropy-based weights in group decision-making with hybrid preference representations. The paper proposes a comprehensive group decision model that combines crisp values with interval data utilizing entropy-based weights.

## 3. The Hellwig’s Method

Hellwig’s method [22,23] allows for determining the ranking of objects under consideration (alternatives) considering multiple variables (criteria) by calculating the distances between the ideal object (ideal solution) and the objects (alternatives). Designed initially for crisp data, this method has been extended to address various problems and scenarios. This extension involves adapting the method to handle situations where the data are not strictly crisp but possess some level of uncertainty or imprecision [46,47,48,49,50]. Hellwig’s method has been applied in multidimensional socio-economic analyses, such as socio-economic development [51,52,53,54], sustainable development [55,56], agriculture development [57,58,59,60], human capital [61], innovation [62], quality of life [47,48,50], and education [63,64]. Hellwig’s method has also been applied to support decisions, for instance, in negotiation [49,50], the Football League [65], and the selection of locations [66].

The general framework of Hellwig’s method is as follows:

We have m objects (alternatives) $A_{1},A_{2},\ldots ,A_{m}\;$and n variables (criteria) $C_{1},C_{2},\ldots ,C_{n}$. In the first step, the data matrix is established:

(13) $$D=\left[x_{ij}\right],$$

where $x_{ij}$ is the value of the j-th variable (criterion) for the i-th object (alternative) i=1,…,m, j=1,…, n.

Next, the vector of weights is determined:

(14) $$w=\left[w_{1},\ldots ,w_{n}\right]$$

where $w_{j}>0\;\left(j=1,\ldots ,\;n\right)$ is the weight of the variable (criterion) $C_{j}$ and $\sum_{j=1}^{n}w_{j}=1$.

It is worth noticing that in the original Hellwig’s framework, equal weights are assumed.

Also, the variables (criteria) are categorized as stimulant (positive) corresponding to benefit criteria, and destimulant corresponding to cost criteria.

The ideal solution I is built using the following equation:

(15) $$I=\left[x_{1}^{+},\ldots ,x_{n}^{+}\right]$$

where:

(16) $$\;\;x_{j}^{+}=\left\{\begin{array}{ll} \underset{i}{\max}x_{ij} & \mathrm{for}\;\mathrm{stimulant}\\ \underset{i}{\min}x_{ij} & \mathrm{for}\;\mathrm{destimulant}\; \end{array}\right.$$

for j=1,…, n.

In the next step, the normalized matrix Z is determined:

(17) $$Z=\left[z_{ij}\right]$$

where $z_{ij}\;$is a normalized value of $x_{ij}$ (i=1,…,m, j=1,…, n).

In the classical Hellwig’s approach, the standardization formula (S) is used to normalize data from the decision matrix, i.e.,

(18) $$z_{ij}=\frac{x_{ij}-{\bar{x}}_{j}}{S_{j}}$$

where ${\bar{x}}_{j}=\frac{1}{m}\sum_{i=1}^{m}x_{ij}$, $S_{j}=\sqrt{\frac{1}{m}\sum_{i=1}^{m}{\left(x_{ij}-{\bar{x}}_{j}\right)}^{2}}$.

Such an approach is utilized in [53,54,55,61,62,67]. The max–min normalization (Formula (12)) is occasionally also used, for instance, in [68,69,70].

In the following step of Hellwig’s algorithm, the weighted normalized matrix, denoted as $\tilde{D},$ is defined as:

(19) $$\tilde{D}=\left[{\tilde{x}}_{ij}\right],$$

where:

(20) $${\tilde{x}}_{ij}=w_{j}z_{ij}$$

Next, the distances of the i-th alternative $A_{i}\;$from the ideal I are calculated using the following formula:

(21) $$d_{i}\left(A_{i},I\right)=\sqrt{\sum_{j=1}^{n}{\left({\tilde{x}}_{ij}-{\tilde{x}}_{j}^{+}\right)}^{2}}$$

where ${\tilde{x}}_{ij},\;{\tilde{x}}_{j}^{+}$ are weighted normalized values $x_{ij}$ and $x_{j}^{+}$, respectively.

The Hellwig’s measure$\;H_{i}$ is determined as follows:

(22) $$H_{i}=1-\frac{d_{i}}{d_{0}}$$

where $d_{0}=\bar{d}+2S$ for $\bar{d}=\frac{1}{m}\sum_{i=1}^{m}d_{i}$, $S=\sqrt{\frac{1}{m}\sum_{i=1}^{m}{\left(d_{i}-\bar{d}\right)}^{2}}.$

Finally, a ranking of objects (alternatives) is provided based on the descending values of $H_{i}.$ The higher the Hellwig’s value, the higher the ranking position for the respective object (alternative).

## 4. A Case Study: Evaluation of Sustainable Development in the Education Area by Hellwig’s Framework

### 4.1. The Source of Data

The 2030 Agenda for Sustainable Development, adopted by all United Nations Member States in 2015, presents a collective roadmap for fostering peace and prosperity for both people and the planet, spanning the present and the future. At its core are the 17 Sustainable Development Goals (SDGs), which serve as a pressing call to action for all nations, irrespective of their development status, to engage in a global partnership [71]. Education is pivotal for economic growth and job creation, as it improves employability, productivity, innovation, and competitiveness. In a broader context, education is also a prerequisite for achieving many other Sustainable Development Goals (SDGs) [72,73]. Monitoring progress on SDG 4, referred to as ‘Quality Education,’ in the EU context, focuses on primary education, higher education, adult learning, and digital skills [63,64,74].

This study aims to assess and compare the implementation of SDG 4 across European Union member states using Hellwig’s method with various entropy-based weights. We employed data from Eurostat for 2021, focusing on Sustainable Development indicators related to education (SDG 4) [75] for this year. Education, as a complex phenomenon, was characterized using five criteria [75]:

- $C_{1}:\;$Early leavers from education and training (%) [sdg_04_10a] (destimulant)
- $C_{2}$: Tertiary educational attainment (%) [sdg_04_20] (stimulant)
- $C_{3}$: Participation in early childhood education (%) [sdg_04_31] (stimulant)
- $C_{4}$: Adult participation in learning in the past four weeks (%) [sdg_04_60] (stimulant)
- $C_{5}$: Share of individuals having at least basic digital skills (%) [sdg_04_70] (stimulant)

The set of indicators for SDG 4 encompasses key aspects intended to monitor progress across diverse educational levels and domains. Table 1 illustrates five indicators measuring the assessment of SDG 4 in EU countries in the year 2021.

### 4.2. Results

To observe the impact of normalization on Hellwig’s results, we designed a comparative study in which we considered a combination of normalization mode for the EWM and normalization formula for Hellwig’s method: combination mode I (CMI): none in the EWM and S in Hellwig’s method; combination mode II (CMII): MM in the EWM and S in Hellwig’s method; combination mode III (CMIII): none in the EWM and MM in Hellwig’s method; combination mode IV (CMIV): MM in the EWM and MM in Hellwig’s method; combination mode V (CMV): none in the EWM and VN in Hellwig’s method; combination mode VI (CMVI): MM in the EWM and VN in Hellwig’s method.

The calculation of the entropy-based weights without normalization (EWMn) and with MM normalization (EWMMM) in Step 2 is presented in Table 2.

The Garuti’s *G* compatibility index [76] is employed for comparing weight systems, and its calculation is as follows [76]:

(23) $$G_{\mathrm{EWMMM}\;}^{\mathrm{EWMn}\;}=\overset{n}{\underset{j=1}{\sum }}\;\left[\frac{\min\left(w_{j}^{\mathrm{EWMn}},\;w_{j}^{\mathrm{EWMMM}}\right)}{\max\left(w_{j}^{\mathrm{EWMn}},w_{j}^{\mathrm{EWMMM}}\right)}\cdot \frac{\left(w_{j}^{\mathrm{EWMn}}+w_{j}^{\mathrm{EWMMM}}\right)}{2}\right]$$

The index G = 1 indicates full compatibility of two systems of weights, while G = 0 signifies total incompatibility. The Garuti index value in our study, $G_{\mathrm{EWMMM}\;}^{\mathrm{EWMn}\;}=0.573$, confirms the weak compatibility of the systems of weights. Let us note that the weight coefficients correspond to their coefficients of variation (see Table 1). The most important criterion is $C_{4}$ (64.73%), followed by $C_{1}$ (40.84%), $C_{2}$ (21.72%), and $C_{5}$ (21.10%) in that order. The least important criterion is $C_{3}$, corresponding to a variability of 8.40%.

**Figure 1:**
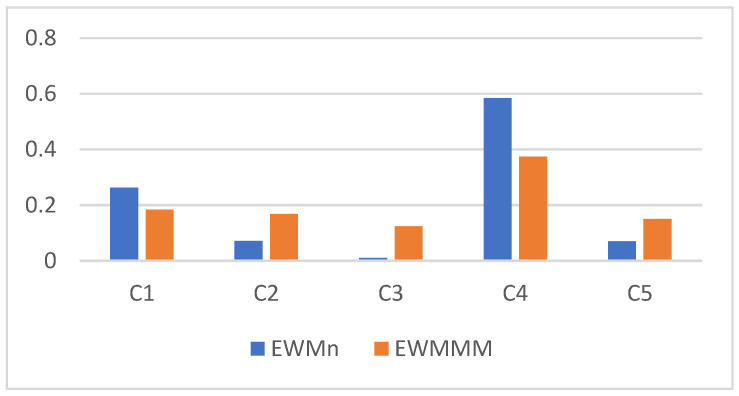
Comparison of two systems of entropy-based weights.

The comparison of two systems of weights is presented in Figure 1. We can observe that the max–min method in the EWMMM resulted in a flattening of the differences between criteria values. The most important criteria (C1, C4) obtained lower weights, while the least important ones (C2, C3, C5) received higher weights compared to the EWMn.

The ideal solution I (Formulas (15) and (16)) has the form $\left[2.40,\;62.60,\;100.00,\;34.70,\;79.18\right].$ The criteria values are normalized using the standardization (Formula (18)), max–min (Formula (12)), and vector normalization (Formula (9)) methods. Next, the values of distance measures between the alternatives (countries) and *I* are calculated (Formula (21)). Finally, Hellwig’s measures with six combination modes are determined (Formula (22)). The Hellwig’s measure values and the rankings of countries obtained by combination modes (two variants of entropy-based weights and three normalization formulas in Hellwig’s method) are presented in Table 3.

While analyzing the positions of the EU countries in the overall rankings obtained using six combination modes of Hellwig’s method, one may observe that the rankings are very similar, as confirmed by the Kendall tau coefficients (Table 4). What is interesting is that the differences between the rankings are one or two positions.

Moreover, the disparities in Hellwig’s values are minimal, as evidenced by Pearson’s coefficients (Table 5).

**Figure 2:**
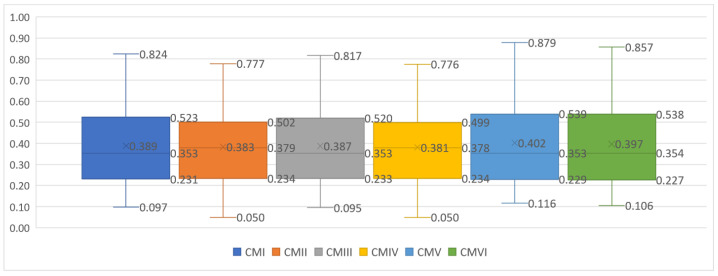
Box plots for the six combination modes of Hellwig’s measures.

Basic descriptive statistics for six combination modes of Hellwig’s measures are presented in Table 3 and Figure 2.

When examining the box plots representing Hellwig’s values, we can observe that the distributions obtained for various combination modes are very similar. The differences in Hellwig’s values among the EU countries range from 0.722 to 0.763. The mean falls between 0.387 and 0.402, with a standard deviation of 0.191 to 0.201. At the same time, no matter which combination mode was used, the results indicate similar significant disparities among EU countries in achieving the SG4 goal (the pattern of differences was preserved). No country excelled or lagged in all criteria. Sweden, the Netherlands, and Finland received the top scores across all Hellwig’s modes among EU countries in 2021. High Hellwig’s scores were also attained by Denmark, Slovenia, Estonia, and Luxembourg. Conversely, Bulgaria, Romania, and Greece recorded the lowest scores in 2021.

It is indeed a surprising finding that using various techniques for data normalization for EWM-based weights and for Hellwig’s algorithm does not cause significant changes in the final evaluation, although it significantly affects the weights. This phenomenon requires deeper consideration and analysis, which we will conduct in the following section.

## 5. Discussion

Chen [17] investigated the impact of max–min normalization on the EWM and the relationships between the EWM and TOPSIS with various normalization approaches in TOPSIS. The studies showed that normalization can influence the decision outcomes of the entropy-based TOPSIS method. Max–min normalization affects the EWM results and fails to represent the raw data’s diversity accurately. The examples presented by Chen show that the system of weights differs in values and the order of importance of criteria. Chen [17] also does not recommend MM for the entropy weight method, and VN is advised for the TOPSIS method. He claims that the weights become meaningless if MM is employed for the EWM.

The original Hellwig approach used equal weights for criteria. Maggino and Ruviglioni [77] observed that equal weights were commonly employed in many applications. Greco et al. [78] argued in favor of equal weights for various reasons, such as simplicity of implementation, the absence of a theoretical foundation to support a differentiated weighting scheme, disagreement among decision-makers, and insufficient statistical or empirical evidence. Therefore, to analyze more deeply the impact weights on the final ranking, we compared the results presented in Table 3 with the results of Hellwig’s method H_S, H_MM, and H_VN for the equal weights and S, MM, and VN normalization formulas, respectively (Table 6).

Similarly, as with entropy-based weights applied in the Hellwig measure (Table 4 and Table 5), the rankings and the disparities in Hellwig’s values obtained by H_S, H_MM, and H_VN with equal weights are similar (Table 7 and Table 8).

Strong Pearson correlations were obtained for different systems of weights and the same data normalization procedure. For S normalization, we obtained the following: P(H_S, CMI) = 0.872, P(H_S, CMII) = 0.936; for MM normalization: P(H_MM, CMIII) = 0.873, P(H_S, CMIV) = 0.994; and for the VN formula: P(H_VN, CMV) = 0.956, P(H_VN, CMVI) = 0.965. The Kendall tau correlation coefficients were not that high but still indicated moderately strong associations, yielding the following results: K(H_S, CMI) = 0.783, K(H_S, CMII) = 0.829; for MM normalization: K(H_MM, CMIII) = 0.772, K(H_MM, CMIV) = 0.812; and for the VN formula: K(H_VN, CMV) = 0.818, K(H_VN, CMVI) = 0.835.

Our study noticed that weights obtained from non-normalization and the MM approach in the EWM are not strongly compatible (Garuti index 0.573). However, they preserve the order of importance of the criteria. It is not unexpected that different objective weighting methods lead to different systems of weights. However, one might be surprised that such distinct systems of weights across three different normalization formulas in Hellwig’s measure result in highly similar rankings (see Table 1 and Table 4) and a very strong correlation between Hellwig’s values (Table 1 and Table 5).

Therefore, we decided to check if this situation is related to the structure of the Eurostat data used for analysis (i.e., whether it is case-specific). The Pearson correlation coefficients between criteria are presented in Table 9.

It is clear that in our case, some countries’ single-criterion performances are moderately to highly correlated. Thus, we decided to check whether this correlation may be considered a factor affecting similarities in ranking despite dissimilarities in weights. We organized two simulation studies, each with two scenarios, that amounted to experimenting with different modifications of Eurostat data.

In Study 1, in each replication (repeated 1000 times), we simulated a data structure similar to data from Table 1, i.e., consisting of 27 alternatives and five evaluation criteria. We sampled the performances of alternatives for each criterion, using the normal distribution observed for each criterion in original Eurostat data and their actual means. Additionally, we enforced the correlations among the single-criterion performances as determined for Eurostat data (see Table 6). In the simulation, we only compared Hellwig’s results obtained for two different setups, CMI and CMII, i.e., those that differ in using (or not) MM normalization when determining weights, keeping the same standardization-based approach in Hellwig’s algorithm. This way, we can observe how the specificity of such non-trivial single-criterion correlations of performances may affect EWM weights and their impact on Hellwig’s rank orders of alternatives.

**Figure 3:**
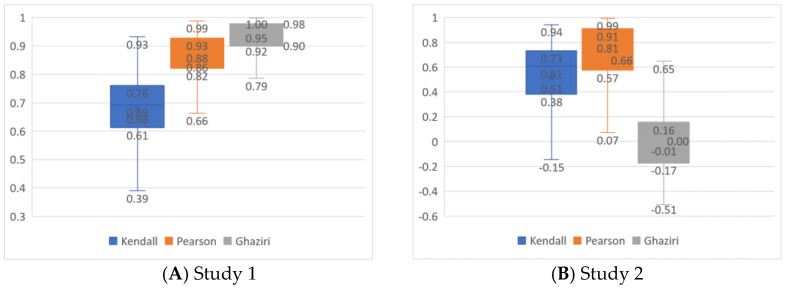
Box plots for correlation coefficients between CMI and CMII results in simulation studies with Ghaziri indexes of quality of sampling.

The results of simulation study 1 show that the correlations between Hellwig’s indexes for CMI and CMII, as well as the resulting rankings, are high. However, they are not as high as in our real-world case of evaluating the EU countries (see Figure 3A).

We may see that the average Pearson coefficient between counties’ performances measured by Hellwig’s index equals 0.863 in simulation. Moreover, even a third quartile (0.93) is smaller than the value we obtained for our case, i.e., 0.987. In fact, the relative frequency of obtaining results that are at least as correlated as in our real-world case is as small as 0.1% (observed in one replication only). The same applies to comparing Kendall ordinal correlations, though the differences are even more visible here. The Garuti index that measures the similarity of two systems of weights for our simulation data equals 0.45. It is not very different from what we observed in empirical data (0.54). However, when we look in detail at the relationships among the weights of subsequent criteria obtained in each iteration for the EWMn and EWMMM methods, we will find that in 13 replications only (1.3%), the order of weights was the same as it was for the EWMn and EWMMM determined for empirical data. The same systems of weights have a Kendall tau index equal to 1. In our simulation, the average Kendall tau was 0.194. To be sure the results we obtain are reliable, i.e., adequately resembling the situation of intercorrelated criteria, we determined the adjusted RV Ghaziri index [79] between the correlation matrices of criteria for data matrices sampled for each iteration and the correlation matrix from Table 6. The Ghaziri index was 0.92, which proves extreme similarity.

From the above, one may conclude that case-specificity may be an issue here and impacts the similarity of rankings despite the dissimilarity of weights. It is, however, an important finding that clearly shows that a higher correlation among the performances of alternatives makes the Hellwig results less sensitive to the criteria weights.

In view of the above results, in Study 2, we decided to relax the requirements for the correlation of criteria within decision matrices. Therefore, we sampled 1000 decision matrices, in which we only ensured that the data for each criterion came from the normal distribution and had the means and standard deviations equal to the empirical ones. Then, we determined the same comparative indexes as for the results in Study 1. Their general distributions are shown in Figure 3B. Here, the differences in Hellwig’s CMI and CMII results seem more evident. The average Kendall and Pearson correlations between CMI and CMII are 0.510 and 0.656, respectively. These correlations are significantly smaller than those obtained in Study 1 (at *p* < 0.001 in the Mann–Whitney test). It clearly shows that the rankings and ratings start to differ for the EWMn and EWMMM-based weights if the correlations do not bind the single-criterion performances. The significant differences between the sampled performance matrices and the empirical ones in terms of correlations of criteria are proven by the distribution of the adjusted RV Ghaziri index (with an average value equal to 0).

Interestingly, in Study 2, the sampled data allowed for generating the systems of weights according to the EWMn and EWMMM, which were more similar than those in Study 1. The average Garuti index in Study 2 equals 0.585, and the Garuti’s distribution is significantly different in Studies 1 and 2, at *p* < 0.001 in the Mann–Whitney test. However, the similarity of the results (rankings or ratings) is still weaker in Study 2. It strengthens our earlier observation that the correlation of the criteria may make the results insensitive to the normalization methods used, no matter how similar or different the EWM weights they produce.

## 6. Conclusions

This research aligns with the broader context of studies related to the impact of certain factors on the final ranking obtained through multiple-criteria decision-making methods. In this study, we addressed how selecting variants of entropy-based weights and normalization formulas influences the ranking obtained through Hellwig’s method. Four primary scientific goals were achieved in this paper.

The first goal was to analyze the impact of two variants of entropy-based weights (with and without max–min normalization) on the outcome obtained by Hellwig’s method. It is important to emphasize that the major advantage of entropy-based weight methods is their ability to handle the lack of knowledge about important criteria through simple and uncomplicated calculations using only information provided by the criteria and an intuitive interpretation of the entropy measure. The second goal of the paper was to analyze the impact of three different normalization methods (standardization, max–min, and vector normalization) on the outcome obtained by Hellwig’s method. The comparative analysis focused on the most commonly used normalization methods in Hellwig’s and other MCDM methods. The third goal was to analyze the impact of the combination of entropy weight methods and normalization formulas (six combination modes) on the outcome obtained by Hellwig’s method. The final goal was to compare the performance of different variants of Hellwig’s method for analyzing sustainable development in the education sector. We compared the rankings obtained by Hellwig’s method using different combination modes.

The study successfully achieved its objectives by comprehensively analyzing the impact of entropy-based weights and different normalization methods on the outcomes derived from Hellwig’s method. This thorough examination provided valuable insights into the effectiveness of various approaches in the decision-making process. The research contributes to the existing literature by offering a unique perspective on the combined use of entropy-based weights and normalization techniques within the context of Hellwig’s method. To the best of our knowledge, no prior studies have explored this aspect to such a degree, making our work a significant contribution to the field of multi-criteria decision-making.

The study also analyzes the impact of both entropy-based weights and normalization methods in evaluating sustainable development in the education area. In summary, the differences between the systems of weights obtained by two entropy-based methods are significant. The impact of the normalization formula on the final ranking obtained by Hellwig’s method while maintaining the weight system is negligible. However, surprisingly, the combination modes of Hellwig’s measure and the normalization formula did not significantly affect the results in our real-world problem, i.e., positioning EU countries in the rankings. In each of the obtained rankings, the countries with the highest levels of realization of SDG goals in the education sector were Sweden, Finland, and the Netherlands, while the lowest-ranked countries were Bulgaria, Romania, and Greece. However, we proved that the lack of significant differences in the EU case is related to the specificity of the problem and the more than average correlation of some criteria in the decision matrix. Our simulation studies showed that the result could be more different when we compare data samples with similar distributions and correlations. If the correlation weakens, the normalization techniques significantly affect differences in Hellwig’s rankings and ratings.

Despite the valuable insights presented in this study, it allowed us to acknowledge some limitations associated with the research design, particularly in the context of the data sample structure. The real-world data may reveal some interdependencies that make the use of different normalization and MCDM techniques lead to similar results. In view of them, an empirical study’s findings may be constrained by the representativeness of the primary data set used (here, the Eurostat records of EU countries’ performances). A more extensive and diverse set of samples could provide a broader understanding of the problem and indicate whether the regularities observed are case-specific only.

Therefore, there is a need for further research to delve into the in-depth analysis of the relationship between the number of alternatives, criteria, and data structure, measured by correlations between criteria. The simulation experiments with different levels of correlations among criteria could provide better-grounded conclusions on how Hellwig’s method performs depending on the version of the EWM applied in advance to produce the system of weight for MCDM analysis. Additionally, exploring criteria of various descriptive statistics (e.g., mean, standard deviation, coefficient of variation, presence of outliers) and examining the resulting weighting systems obtained through entropy methods and the consistency of rankings obtained by other multiple criteria methods alternative to Hellwig’s, such as TOPSIS or VIKOR, could tell us more about which of these techniques is more resistant to the peculiar patterns of the correlations among the evaluation criteria.

## Figures and Tables

**Table 1:** Indicators measuring the assessment of SDG 4 in EU countries in the year 2021.

EU Country	$C_{1}$	$C_{2}$	$C_{3}$	$C_{4}$	$C_{5}$
Austria	8.0	42.4	89.0	14.6	63.33
Belgium	6.7	50.9	97.9	10.2	54.23
Bulgaria	12.2	33.6	79.4	1.8	31.18
Croatia	2.4	35.7	77.8	5.1	63.37
Cyprus	10.2	58.3	85.8	9.7	50.21
Czechia	6.4	34.9	84.2	5.8	59.69
Denmark	9.8	49.7	97.0	22.3	68.65
Estonia	9.8	43.2	91.5	18.4	56.37
Finland	8.2	40.1	90.6	30.5	79.18
France	7.8	50.3	100.0	11.0	61.96
Germany	12.5	36.9	93.1	7.7	48.92
Greece	3.2	44.2	68.8	3.5	52.48
Hungary	12.0	32.9	93.4	5.9	49.09
Ireland	3.3	61.7	96.4	13.6	70.49
Italy	12.7	28.3	91.0	9.9	45.60
Latvia	7.3	45.5	94.5	8.6	50.80
Lithuania	5.3	57.5	92.1	8.5	48.84
Luxembourg	9.3	62.6	88.9	17.9	63.79
Malta	10.7	42.5	86.2	13.9	61.23
Netherlands	5.1	55.6	93.0	26.6	78.94
Poland	5.9	40.6	90.4	5.4	42.93
Portugal	5.9	47.5	90.5	12.9	55.31
Romania	15.3	23.3	75.6	4.9	27.82
Slovakia	7.8	39.5	77.4	4.8	55.18
Slovenia	3.1	47.9	92.3	18.9	49.67
Spain	13.3	48.7	96.0	14.4	64.16
Sweden	8.4	49.3	96.1	34.7	66.52
Min	2.40	23.30	68.80	1.80	27.82
Max	15.30	62.60	100.00	34.70	79.18
Mean	8.24	44.58	89.22	12.65	56.29
Standard deviation	3.37	9.68	7.50	8.19	11.88
Coefficient of variation	40.84	21.72	8.40	64.73	21.10

Source: Eurostat [SDG 4] [75]. Data for Greece for criterion $C_{3}$ were estimated.

**Table 2:** Entropy-based weights obtained using different formulas.

Variants of EWM	$C_{1}$	$C_{2}$	$C_{3}$	$C_{4}$	$C_{5}$
EWMn	0.263	0.072	0.011	0.584	0.070
EWMMM	0.184	0.168	0.124	0.374	0.150

Source: Authors’ calculations.

**Table 3:** The values and rank-ordering of EU countries obtained by the combination modes of Hellwig’s measures.

Country	CMI	Rank CMI	CMII	Rank CMII	CMII	Rank CMII	CMIV	Rank CMIV	CMV	Rank CMV	CMVI	Rank CMVI
Austria	0.454	9	0.451	9	0.452	9	0.449	9	0.462	8	0.459	8
Belgium	0.353	14	0.379	14	0.353	14	0.378	14	0.353	14	0.354	14
Bulgaria	0.097	27	0.066	26	0.095	27	0.067	26	0.116	27	0.106	27
Croatia	0.231	21	0.232	22	0.233	21	0.232	22	0.225	22	0.225	22
Cyprus	0.315	16	0.331	16	0.313	16	0.329	16	0.327	15	0.326	15
Czechia	0.240	20	0.244	18	0.241	19	0.243	18	0.239	20	0.238	20
Denmark	0.615	4	0.616	4	0.610	4	0.610	4	0.643	4	0.637	4
Estonia	0.525	6	0.502	7	0.521	6	0.499	7	0.548	6	0.539	6
Finland	0.794	2	0.712	3	0.788	2	0.708	3	0.839	2	0.813	2
France	0.367	13	0.400	13	0.367	13	0.397	13	0.370	13	0.372	13
Germany	0.241	19	0.238	20	0.237	20	0.234	21	0.264	19	0.257	19
Greece	0.190	25	0.182	25	0.192	25	0.185	25	0.183	25	0.185	25
Hungary	0.202	24	0.197	24	0.199	24	0.193	24	0.221	23	0.214	23
Ireland	0.454	8	0.498	8	0.455	8	0.496	8	0.448	9	0.453	9
Italy	0.287	18	0.243	19	0.283	18	0.240	19	0.318	16	0.302	18
Latvia	0.308	17	0.324	17	0.308	17	0.324	17	0.309	18	0.309	17
Lithuania	0.315	15	0.342	15	0.316	15	0.343	15	0.312	17	0.314	16
Luxembourg	0.523	7	0.538	6	0.520	7	0.534	6	0.539	7	0.538	7
Malta	0.410	11	0.405	12	0.407	11	0.400	11	0.431	10	0.425	10
Netherlands	0.777	3	0.777	1	0.776	3	0.776	1	0.781	3	0.781	3
Poland	0.231	22	0.234	21	0.232	22	0.236	20	0.229	21	0.227	21
Portugal	0.425	10	0.432	10	0.425	10	0.433	10	0.425	12	0.424	11
Romania	0.135	26	0.050	27	0.130	26	0.050	27	0.177	26	0.154	26
Slovakia	0.209	23	0.210	23	0.209	23	0.210	23	0.210	24	0.209	24
Slovenia	0.585	5	0.556	5	0.586	5	0.560	5	0.585	5	0.579	5
Spain	0.390	12	0.409	11	0.383	12	0.400	12	0.426	11	0.421	12
Sweden	0.824	1	0.775	2	0.817	1	0.771	2	0.879	1	0.857	1
Mean	0.389		0.383		0.387		0.381		0.402		0.397	
SD	0.194		0.192		0.193		0.191		0.201		0.198	
Min	0.097		0.050		0.095		0.050		0.116		0.106	
Max	0.824		0.777		0.817		0.776		0.879		0.857	

Source: Authors’ calculations.

**Table 4:** Kendall tau coefficients between rankings obtained by six combination modes of Hellwig’s measures.

Kendall Tau Coefficient	Rank CMI	Rank CMII	Rank CMIII	Rank CMIV	Rank CMV	Rank CMVI
Rank CMI	1.000					
Rank CMII	0.954	1.000				
Rank CMIII	0.994	0.960	1.000			
Rank CMIV	0.954	0.989	0.960	1.000		
Rank CMV	0.954	0.920	0.949	0.920	1.000	
Rank CMVI	0.972	0.937	0.966	0.937	0.983	1.000

**Table 5:** Pearson coefficients between rankings obtained by six combination modes of Hellwig’s measures.

Pearson Coefficient	CMI	CMII	CMIII	CMIV	CMV	CMVI
CMI	1.0000					
CMII	0.9874	1.0000				
CMIII	0.9999	0.9884	1.0000			
CMIV	0.9874	0.9999	0.9885	1.0000		
CMV	0.9968	0.9757	0.9958	0.9751	1.0000	
CMVI	0.9987	0.9831	0.9980	0.9825	0.9993	1.0000

**Table 6:** The values and rank-ordering of EU countries obtained by the equal weights of Hellwig’s measures.

Country	H_S	Rank H_S	H_MM	Rank H_MM	H_VN	Rank H_VN
Austria	0.467	11	0.462	11	0.445	9
Belgium	0.476	10	0.475	10	0.374	13
Bulgaria	0.022	26	0.026	26	0.056	26
Croatia	0.278	20	0.279	20	0.263	18
Cyprus	0.363	17	0.360	17	0.310	17
Czechia	0.303	18	0.301	18	0.259	19
Denmark	0.595	4	0.582	4	0.570	5
Estonia	0.456	12	0.451	12	0.475	8
Finland	0.588	5	0.580	5	0.693	3
France	0.506	8	0.500	8	0.387	11
Germany	0.245	21	0.236	22	0.202	24
Greece	0.179	25	0.186	24	0.226	21
Hungary	0.214	23	0.206	23	0.170	25
Ireland	0.647	3	0.644	3	0.501	7
Italy	0.179	24	0.172	25	0.213	23
Latvia	0.400	15	0.400	14	0.321	16
Lithuania	0.439	13	0.445	13	0.346	14
Luxembourg	0.548	6	0.540	6	0.509	6
Malta	0.388	16	0.379	16	0.379	12
Netherlands	0.776	1	0.773	1	0.773	1
Poland	0.294	19	0.300	19	0.244	20
Portugal	0.480	9	0.482	9	0.437	10
Romania	−0.107	27	−0.107	27	0.024	27
Slovakia	0.239	22	0.240	21	0.226	22
Slovenia	0.530	7	0.540	7	0.570	4
Spain	0.415	14	0.395	15	0.341	15
Sweden	0.672	2	0.664	2	0.729	2
Mean	0.392		0.389		0.372	
SD	0.196		0.195		0.186	
Min	−0.107		−0.107		0.024	
Max	0.776		0.773		0.773	

Source: Authors’ calculations.

**Table 7:** Kendall tau coefficients between rankings obtained by Hellwig’s measures with equal weights.

Kendal Tau Coefficient	Rank H_S	Rank H_MM	Rank H_VN
Rank H_S	1.000		
Rank H_MM	0.983	1.000	
Rank H_VN	0.840	0.846	1.000

**Table 8:** Pearson coefficients between rankings obtained by Hellwig’s measures with equal weights.

Pearson Coefficient	H_S	H_MM	H_VN
H_S	1.000		
H_MM	0.999	1.000	
H_VN	0.946	0.946	1.000

**Table 9:** Pearson correlation coefficients between criteria.

Pearson Coefficient	$C_{1}$	$C_{2}$	$C_{3}$	$C_{4}$	$C_{5}$
$C_{1}$	1.000				
$C_{2}$	−0.437	1.000			
$C_{3}$	0.037	0.452	1.000		
$C_{4}$	−0.075	0.411	0.506	1.000	
$C_{5}$	−0.383	0.520	0.393	0.706	1.000

## Data Availability

Data are contained within the article.

## Abbreviations

The following abbreviations are used in this manuscript:

AHP	analytic hierarchy process
CMI	combination mode I: none in EWM and S in Hellwig’s method
CMII	combination mode II: MM in EWM and S in Hellwig’s method
CMIII	combination mode III: none in EWM and MM in Hellwig’s method
CMIV	combination mode IV: MM in EWM and MM in Hellwig’s method
CMV	combination mode V: none in EWM and VN in Hellwig’s method
CMVI	combination mode VI: MM in EWM and VN in Hellwig’s method
CRITIC	CRiteria Importance Through Inter-criteria Correlation
DM	decision maker
EWM	entropy weight method
EWMn	entropy weight method without max–min normalization
EWMMM	entropy weight method with max–min normalization
MCDM	multi-criteria decision-making
MM	max–min normalization
MORRA	multi-objective optimization based on the ratio analysis
S	standardization
SD	standard deviation
SDG	Sustainable Development Goal
SN	sum normalization
TODIM	An acronym in Portuguese for Interactive and Multi-criteria Decision Making
TOPSIS	Technique for Ordering Preferences by Similarity to Ideal Solution
VN	vector normalization
